# Time after time – circadian clocks through the lens of oscillator theory

**DOI:** 10.1002/1873-3468.70257

**Published:** 2026-01-21

**Authors:** Marta del Olmo, Carolin Ector, Hanspeter Herzel

**Affiliations:** ^1^ Institute for Theoretical Biology Humboldt Universität zu Berlin and Charité Universitätsmedizin Berlin Germany; ^2^ Springer Nature AG & Co. KG aA Berlin Germany; ^3^ Charité Comprehensive Cancer Center Charité Universitätsmedizin Berlin Germany; ^4^ The Francis Crick Institute London UK

**Keywords:** circadian rhythms, coupled oscillators, coupling, entrainment, limit cycles, oscillator theory, synchronisation

## Abstract

Biological systems are fundamentally rhythmic, with oscillations emerging at multiple scales, from intracellular gene circuits to organ‐level coordination. Many of these rhythms, including the circadian clock, arise from feedback‐driven genetic networks that interact to produce coherent temporal organisation. In this review, we examine the circadian system as a model for understanding the dynamics of coupled biological oscillators. We introduce the core theoretical concepts of delayed feedback, nonlinearity and coupling, and show how these principles govern the emergence of synchronisation, entrainment, and complex dynamics across cellular populations and tissues. Drawing on tools from nonlinear dynamics, we explore how oscillator models help explain robustness, plasticity, and failure modes in circadian systems. Finally, we discuss how this theoretical framework informs experimental design and translational applications in circadian medicine, from optimising drug timing to understanding rhythm disruptions in disease.

## Graphical abstract

Oscillator theory bridges physics and circadian biology. Damped oscillators require external drivers, while limit cycles emerge from delayed feedback and nonlinearities. Coupling enables tissue‐level coherence, and entrainment aligns internal clocks with environmental cues. These principles provide a unified framework for understanding circadian rhythms and their applications in medicine, including chronotherapy.

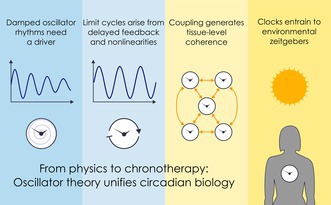

## Abbreviations

**ATP**, adenosine triphosphate

**DDE**, delay differential equation

**FASPS**, familial advanced sleep phase syndrome

**GABA**, gamma‐aminobutyric acid

**ODE**, ordinary differential equation

**PRC**, phase response curve

**PTC**, phase transition curve

**SCN**, suprachiasmatic nucleus

**TTFL**, transcription‐translation feedback loop

**VIP**, vasoactive intestinal peptide

## Symbols

*A*, amplitude

*α*, amplitude relaxation rate

*ɛ*, twist

*F*, zeitgeber strength

*γ*, damping rate

*K*, coupling strength

*r*, radius

*T*, zeitgeber period

*τ*, oscillator's intrinsic period

*φ*, phase

*ψ*, phase of entrainment

*ω*, angular frequency

## Biological timekeepers: a network perspective

Life is rhythmic. From the beating of the heart to the cyclical expression of genes, oscillations are a defining feature of biological systems. Among these, circadian clocks, with their near 24‐h periodicity, stand out for their ubiquity and complexity [1, 2]. Nearly every cell harbours a molecular clock, yet these clocks rarely operate in isolation. Instead, they form hierarchical networks of coupled oscillators that coordinate timekeeping across cells, tissues, and organs [3, 4], and ensure robustness and synchronisation with the environment [5, 6].

In this review, we present a conceptual framework grounded in oscillator theory to understand how circadian rhythms emerge, persist and synchronise. Moving beyond molecular feedback loops, we build on principles from nonlinear dynamics to introduce oscillator types. We then explore how coupling between oscillators shapes collective behaviour, ranging from phase‐locking to complex synchrony and chaos, and how these networks entrain to environmental cycles such as light, temperature and feeding.

Understanding the dynamics of these coupled systems offers insights into long‐standing biological phenomena such as chronotypes, tissue‐specific rhythms, and entrainment ranges. These dynamics also have practical implications: emerging fields like cancer chronotherapy exploit circadian principles to optimise the timing of treatments, leveraging the interplay between internal clocks, cell fate decisions, and drug efficacy.

Rather than providing an exhaustive survey of molecular circadian mechanisms, we aim to highlight unifying dynamical principles that span systems. By doing so, we offer tools for both interpreting complex experimental data and guiding future research at the interface of theory, physiology and medicine.

## Snapshots from oscillator theory: from damped springs to living rhythms

### On the shoulders of giants: from physics to physiology

The development of oscillator theory spans centuries, bridging classical physics and modern biology. Early pioneers focused on basic principles of motion: Newton described how restoring forces govern springs and pendulums [7], providing a mathematical framework for periodic motion. Later, Huygens observed that two pendulum clocks placed on the same surface gradually began to spontaneously synchronise [8, 9], revealing that oscillators could interact and influence each other's timing. Building on these insights, Poincaré explored the complex behaviour of nonlinear systems, including the stability of planetary motion [10, 11]. In doing so, he anticipated concepts like *deterministic chaos*, where systems governed by precise laws can nonetheless exhibit extreme sensitivity to initial conditions, leading to unpredictable dynamics. Although first developed in the context of celestial mechanics, these ideas are now essential for understanding irregularities, transitions, and stability in biological rhythms. Together, their work laid the groundwork for classifying dynamic behaviours and uncovering the fundamental design principles of oscillatory systems.

The 20th century saw further refinement through the work of Duffing and Van der Pol, who introduced nonlinear oscillator models and analysed their responses to periodic forcing [12, 13]. Their studies revealed key phenomena such as resonance, entrainment and bifurcations, highlighting how small changes in parameters could qualitatively alter oscillatory behaviour. The formal theory of coupled oscillators matured through contributions from Andronov, Kuramoto and Pikovsky, among others, who provided a mathematical framework to understand synchronisation across networks [14, 15, 16, 17].

But oscillatory behaviour is not limited to mechanics. It is ubiquitous in biology, from the rhythmic contraction of the heart or hormonal cycles to population‐level rhythms in predator–prey dynamics, tidal rhythms, or vocal fold vibrations. These observations inspired the development of mathematical models across physiology and ecology during the 20th century, including the classic work of Lotka and Volterra on ecological oscillations [18, 19, 20], and Selkov's model of glycolytic feedback [21].

All of these early models paved the way for the application of oscillator theory to circadian biology. From the 1960s onward, mathematical modeling became an integral part of chronobiological research. Wever applied oscillator theory [22] to interpret Aschoff's bunker experiments [23], in which volunteers lived for weeks in isolation from external time cues, revealing the endogenous nature and intrinsic period of human circadian rhythms. Kronauer later developed nonlinear models of human sleep–wake rhythms that captured experimental observations [24]. The revolution of molecular biology introduced transcription‐translation feedback loops (TTFL) as the main paradigm for circadian rhythm generation [1, 2, 25]. Negative feedback loop models pioneered by Ruoff [26], Goldbeter [27] and Forger [28], linked gene regulatory circuits to circadian rhythms in flies and mammals. More recent efforts have focused on specific core clock genes [29, 30, 31], and have also integrated mutant phenotypes [29, 32], gene expression data [33, 34], and multi‐omics profiles [35], producing mechanistically grounded models of circadian timekeeping. Recognising the complex architecture of clocks as coupled oscillator networks, especially in the suprachiasmatic nucleus (SCN) [36, 37, 38, 39, 40] and peripheral tissues [3, 4, 41], recent theoretical research has emphasised the importance of intercellular coupling and spatial organisation in rhythm generation [42, 43, 44, 45, 46, 47]. The discovery of circadian rhythms in cells without TTFL [48, 49] stimulated the development of models to explain the underlying principles that generate circadian rhythmicity in the cyanobacterial ‘phosphorylator’ [30, 50], and in red blood cells through redox oscillations [51].

Given the wealth of mathematical models for circadian rhythm generation, we now focus on the fundamental principles underlying oscillator dynamics: damping, resonance, self‐sustained rhythms, synchronisation and entrainment, and the conditions that give rise to them.

### Damped oscillators and resonances: rhythms need a driver

**Fig. 1:**
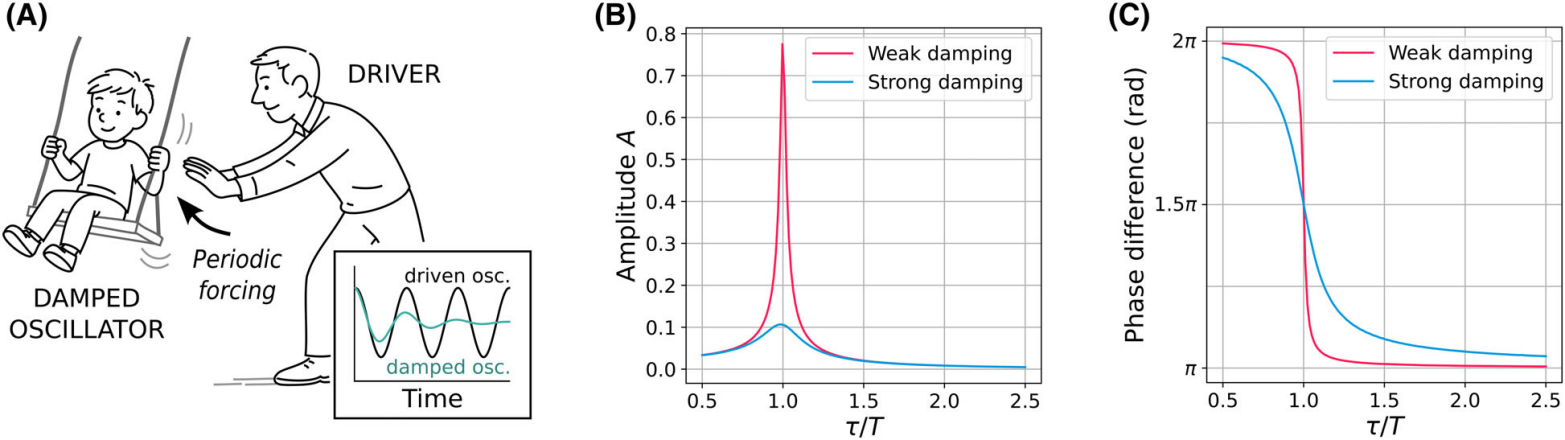
Periodic forcing of damped oscillators with different damping strengths leads to amplitude resonance and pronounced phase shifts. (A) Schematic of a damped oscillator (child on a swing) driven by a periodic force (adult). (B) The amplitude of the driven oscillator increases due to resonance when the forcing period (*T*) approaches the intrinsic period of the damped oscillator (*τ*). Weaker damping results in larger amplitude responses. (C) The phase of the driven oscillator relative to the forcing signal exhibits steep changes near resonance ($\frac{\tau }{T}∼1$), with weakly‐damped oscillators showing larger phase shifts. Across resonance, the phase relationship shifts by 180° (π rad).

Many oscillatory systems, both in physics and biology, can be described as damped oscillators: systems in which energy losses through friction (Fig. 1A), molecular degradation, or other dissipative processes cause spontaneous oscillations to fade, unless energy is continually supplied (Box 1). Biological examples include molecular vibrations, echolocation clicks in dolphins, and circadian rhythms in isolated cells that lack robust transcriptional feedback.

Box 1 **Newton's equation and periodically forced damped oscillators**

Biological oscillators often resemble damped harmonic systems in physics, where intrinsic feedback mechanisms alone are insufficient to sustain rhythms. For example, some clock mutants or cyanobacteria lacking sufficient energy supply fail to maintain circadian oscillations without periodic stimulation [52, 53]. These systems require external inputs (such as light–dark cycles, nutrients, or temperature cycles, among others) to rescue or amplify rhythmic behaviour.

The classical mechanical model capturing such behaviour is the forced, damped harmonic oscillator, governed by Newton's second law [7]. In this model, a restoring force proportional to displacement (Hooke's law [54]) provides negative feedback pulling the system toward equilibrium, while damping represents friction or energy loss causing oscillations to decay over time. The external periodic force mimics environmental inputs driving the oscillator.

Mathematically, the oscillator's displacement *x*(*t*) satisfies:

$$\frac{d^{2}x}{{\mathrm{dt}}^{2}}+2\gamma \frac{\mathrm{dx}}{\mathrm{dt}}+\omega_{0}^{2}x=F_{0}\cos\left(\mathrm{\omega t}\right)$$

where *ω*_0_ is the natural angular frequency determined by the restoring force, *γ* is the damping coefficient quantifying energy loss, and $F_{0}\cos\left(\mathrm{\omega t}\right)$ represents a periodic external force. Angular frequency and period are related by $\omega =\frac{2\pi }{\tau }$.

The asymptotic response to forcing has the form:

$$x\left(t\right)=A\left(\omega \right)\cos\left(\omega \;t-\psi \left(\omega \right)\right),$$

with amplitude $A\left(\omega \right)$ and phase lag between the driver and driven oscillator $\psi \left(\omega \right)$ given by

$$A\left(\omega \right)=\frac{F_{0}}{\sqrt{{\left(\omega_{0}^{2}-\omega^{2}\right)}^{2}+{\left(2\gamma \omega \right)}^{2}}},\;\tan\psi \left(\omega \right)=\frac{2\gamma \omega }{\omega_{0}^{2}-\omega^{2}}.$$

The amplitude *A* peaks near the natural frequency *ω*_0_, a phenomenon known as resonance, where energy absorption is maximised. Near resonance, the phase lag between the driver and the driven oscillator *ψ* changes rapidly, indicating a sharp shift in timing between input and response (Fig. 1).

This behaviour provides a theoretical basis for biological entrainment phenomena: small mismatches between intrinsic circadian periods and environmental cycles can produce disproportionately large shifts in phase [22, 55, 56], underlying variations in chronotype and activity timing [57, 58, 59]. This will be further discussed in the section dedicated to entrainment.

In single cells, circadian oscillations can often be modeled as stochastically driven damped oscillators [60], where molecular noise sustains transient rhythmicity. The characteristic decay time, inversely related to the damping coefficient *γ*, quantifies how quickly oscillations fade in the absence of driving or feedback, highlighting the importance of sustained energy input for persistent rhythms.

In such systems, periodic external inputs (such as light cycles for circadian clocks) can act as drivers (i.e. energy suppliers) that maintain or enhance rhythmic activity through a phenomenon known as *resonance*. When the frequency of the external driver aligns with the oscillator's natural frequency, resonance leads to amplification of the oscillator's amplitude. Weakly damped oscillators act as resonators, selectively responding to specific temporal signals: the weaker the damping, the sharper the resonance peak becomes (Fig. 1B). In strongly nonlinear oscillators however, the single‐peaked resonance curve can deform and admit two stable solutions for the same driving frequency. Classic examples are Duffing‐type systems, which can display birhythmicity [61, 62]: for a given forcing, the oscillator can settle into either a small‐ or a large‐amplitude rhythm. Sweeping the driving frequency can therefore cause abrupt amplitude jumps and hysteresis, phenomena well known in nonlinear dynamics [12, 63].

Beyond the amplitude, resonance also strongly affects the phase relationship between the driver and the driven oscillator. Near resonance, small changes in the input frequency cause rapid shifts in the phase difference between them (Fig. 1C). These steep changes in phase arise naturally in systems with low damping (i.e. strong resonators) [22, 55]. In such systems, the resonance peak is sharp, and as a result, even small changes in the frequency can cause large shifts in the phase difference between the driver and the oscillator (Fig. 1C). This high phase sensitivity is a hallmark of strong resonators and plays an important role in how oscillators respond to external periodic inputs.

### Limit cycles: self‐sustained rhythms through delays and nonlinearities

Unlike damped oscillators, which require ongoing external input to sustain rhythmicity, limit cycle oscillators generate persistent, self‐sustained rhythms autonomously. Their periods and amplitudes are determined by intrinsic system properties, rather than external drivers. In circadian clocks, the energy which is needed to sustain rhythmicity is provided by continuous cellular metabolism: ATP production powers transcription, translation, protein turnover, and post‐translational modifications within the clock network. These processes, together with nonlinear feedback loops, counteract dissipation and stabilise the oscillation. Mathematically, limit cycles correspond to stable periodic solutions of nonlinear dynamical systems [64], with circadian clocks serving as canonical biological examples (see Box 2 for an illustration using the Poincaré oscillator, a simple mathematical model that captures the essential limit cycle properties). Beyond circadian clocks, other biological rhythms exhibit limit cycle dynamics. Examples include cardiac oscillations (with an average heart rate around 70 beats per minute) [65], respiratory rhythms (12–20 breaths per minute) [66], or vocal fold vibrations (period of about 5 ms) [67].

Box 2 **The Poincaré oscillator: a geometric view of self‐sustained rhythms**

To illustrate the geometry of limit cycles, a simple system is the Poincaré oscillator in polar coordinates (*r*,*φ*):

$$\frac{\mathrm{dr}}{\mathrm{dt}}=\mathrm{\alpha r}\left(A-r\right),\;\frac{\mathrm{d\varphi}}{\mathrm{dt}}=\omega$$

Here, *A* represents the limit cycle amplitude, *r*(*t*) represents the radial coordinate (i.e. instantaneous amplitude) and *φ*(*t*) the phase of the oscillator. The radial component $\frac{\mathrm{dr}}{\mathrm{dt}}$ ensures that trajectories converge to a stable limit cycle of radius *r* = *A*, regardless of the initial amplitude. Meanwhile, the angular velocity $\frac{\mathrm{d\varphi}}{\mathrm{dt}}=\omega \equiv \frac{2\pi }{\tau }$ remains constant, resulting in a steady, regular advancement of the phase. This model exhibits two fundamental properties of biological oscillators: amplitude perturbations decay back to the cycle at a rate determined by *α*, and the phase advances steadily, independent of amplitude, reflecting the oscillator's internal timekeeping.

Although very simple, the Poincaré oscillator captures the essence of amplitude‐phase models, which offer a general and intuitive abstraction of rhythmic systems. These models describe oscillatory dynamics without reference to specific molecular details, instead relying on two variables (radius *r* and phase *φ*) to characterise system behaviour. Their simplicity allows for geometric intuition and analytical tractability, making them particularly useful for studying generic oscillator properties such as entrainment, phase‐locking, or synchronisation across coupled systems.

One particularly relevant parameter in this model is the amplitude relaxation rate *α*, which quantifies how quickly a disturbed oscillator returns to its limit cycle. A small *α* results in slow relaxation and long memory of perturbations, while a large *α* implies a strongly attracting cycle with rapid stabilisation. This concept is crucial when analysing how biological clocks respond to transient stimuli or phase‐shifting cues.

The Poincaré oscillator is autonomous in its basic form: it oscillates without requiring any external input. However, it can be extended to include periodic forcing, such as a sinusoidal zeitgeber, to study entrainment. This extension makes the model particularly relevant in circadian biology, where it has been used to explore phase response curves [68], entrainment ranges [37, 55], and network behaviour in coupled oscillator systems [43, 69, 70, 71]. In fact, amplitude‐phase models like this have been widely used in chronobiology to study fundamental properties of circadian oscillators in both single cells and tissues, offering insights that complement more detailed molecular models.

An important extension of the Poincaré oscillator is the inclusion of *twist*, a dependency between amplitude and period. In the basic model, the angular velocity *ω* is constant and independent of amplitude. However, in many biological systems, the period can shift slightly with changes in amplitude [44, 72]. This effect can be captured by making *ω* a function of *r*, such as

$$\omega \left(r\right)=\omega_{0}+\epsilon \left(1-r\right),$$

where *ɛ* quantifies the strength of the twist. Including twist into Poincaré oscillators introduces richer dynamical behaviour, particularly under external forcing, and has been shown to influence entrainment range, phase response curves, and response of oscillators to coupling [44, 73, 74].

**Fig. 2:**
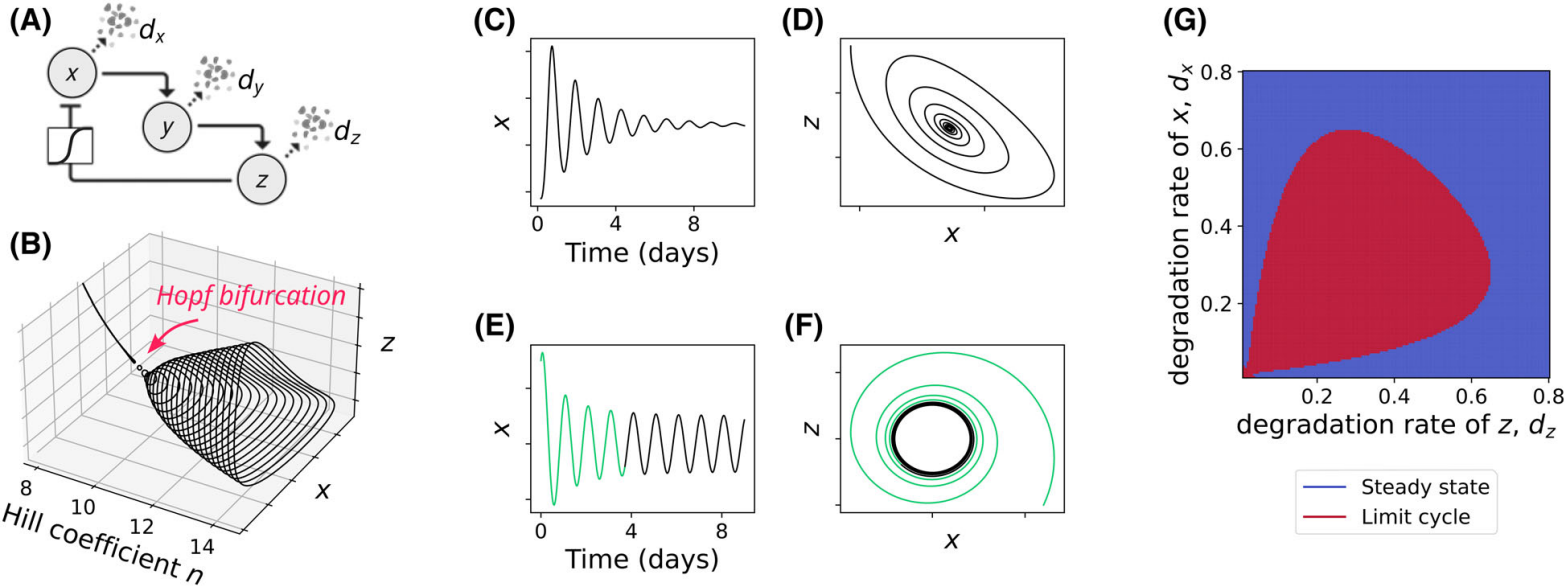
Emergence of limit cycle oscillations *via* a Hopf bifurcation in the three‐variable Goodwin model. (A) Schematic of the Goodwin model for circadian limit cycle oscillations. Variable *z* represses *x* through a nonlinear, switch‐like function with the sharpness of the switch being characterised by a Hill coefficient. (B) One‐parameter bifurcation diagram showing the effect of increasing the Hill coefficient (*n*). Stronger nonlinearity (higher *n*) leads to a Hopf bifurcation and the onset of self‐sustained oscillations, as indicated by the emergence of a closed trajectory for *n* > 9. Results are obtained for numerical integration of the Goodwin model [77] for the following parameter values: *d*_*x*_ = 0.2, *d*_*y*_ = 0.15, *d*_*z*_ = 0.1. (C, D) For *n* < 9, the system displays damped oscillations that converge to a stable fixed point (C, time series; D, phase portrait of *x* versus *z*). (E, F) For *n* > 9, the system exhibits self‐sustained limit cycle oscillations (E, time series; F, phase portrait of *x* versus *z*). Transients (i.e. the initial trajectories before the system settles onto the limit cycle) are shown in green. Regardless of the initial conditions, the trajectories converge to the same stable limit cycle. (G) Two‐parameter bifurcation diagram showing regions of parameter space that give rise to either steady states or limit cycles, depending on combinations of degradation rates of *x* (*d*_*x*_) and *z* (*d*_*z*_).

At the molecular level, circadian limit cycles emerge from delayed negative feedback loops and nonlinear regulatory interactions [75] (networks akin to Fig. 2A), the core features of transcription‐translation feedback loops (TTFLs) [1]. As a minimal mathematical illustration, in Fig. 2 we show simulations of the Goodwin model [76], a classic feedback oscillator in which a gene product indirectly represses its own expression through a delayed negative feedback loop (Fig. 2A).

To demonstrate that these rhythms are generated endogenously rather than driven by the environment, organisms are studied under constant conditions. This experimental approach dates back to the 18th century, when Jean‐Jacques de Mairan observed that the leaves of *Mimosa pudica* plants continued their daily opening and closing in complete darkness [78]. Two centuries later, chronobiology pioneers such as Aschoff, Pittendrigh and Kleitman systematically demonstrated that circadian rhythms persist in the absence of environmental timing cues (zeitgebers), even in humans isolated in bunkers or caves [5, 6, 23, 79, 80], thus providing compelling evidence that biological clocks behave as endogenous limit cycle oscillators.

A common route by which oscillations arise in biological systems is through a Hopf bifurcation [64, 81], in which a stable fixed point loses stability and gives rise to a stable limit cycle (Fig. 2B). Such dynamics are characterised by autonomous periods and amplitudes. They can be visualised both as time series (Fig. 2C,E) and geometrically in the ‘phase space’ (Fig. 2D,F) of relevant variables (e.g. concentrations of clock proteins and transcripts). From arbitrary initial conditions, the system typically shows transient, non‐periodic behaviour (green traces in Fig. 2E,F) before settling onto the periodic orbit. Over time, all trajectories converge to the closed curve representing the limit cycle (Fig. 2F). Limit cycles therefore act as attractors [64]: states that originate from a broad range of initial conditions eventually evolve into the same stable rhythm.

From a dynamical systems perspective, the behaviour of biological oscillators depends on phase space variables (such as transcript and protein concentrations) and parameters (such as temperature, ATP levels, or cell size). Many parameter combinations lead to stable steady states through damped oscillations (Fig. 2C,D), corresponding to rhythms that decay over time. As parameters are gradually varied, self‐sustained oscillations can emerge through a Hopf bifurcation. When two parameters are varied together, the set of Hopf bifurcation points often forms a closed boundary in parameter space. Inside this boundary, sometimes visualised as a ‘Hopf bubble’, the system exhibits stable limit cycle oscillations, whereas outside of it, the dynamics return to steady states (Fig. 2G).

#### Wanted: delays!

Feedback delays are essential for sustaining oscillations in transcription‐translation feedback loops (TTFLs) [69, 81, 82]. Theoretical studies suggest that rhythmicity can only be maintained if the feedback delay is sufficiently long (see Box 3), typically around one‐quarter of the oscillator's period [66, 77, 82, 83]. For circadian clocks with 24‐h cycles, this translates to delays of at least 6 h, which far exceed the duration of basic transcription and translation steps. This mismatch indicates that additional regulatory mechanisms must operate to effectively prolong the delay in circadian systems.

Box 3 **Delay differential equations**

Self‐sustained oscillations, such as those seen in circadian clocks, require two essential ingredients: sufficient delay and strong nonlinearity. These features are captured in a classic one‐variable delay differential equation (DDE):

$$\frac{\mathrm{dx}\left(t\right)}{\mathrm{dt}}=\frac{a}{1+x{\left(t-\tau \right)}^{n}}-\mathrm{bx}\left(t\right)$$

In this equation, *x* represents a transcriptional repressor that inhibits its own production with a time delay *τ*. The parameter *a* denotes the maximal production rate of the repressor (i.e. the rate of synthesis in the absence of repression), and *b* represents the degradation rate. The first term on the right‐hand side $\left(\frac{a}{1+x{\left(t-\tau \right)}^{n}}\right)$ describes production under delayed negative feedback, with nonlinearity introduced via a Hill function with coefficient *n*. The second term ($-\mathrm{bx}\left(t\right)$) captures linear degradation in the model equations. The delayed argument $x\left(t-\tau \right)$ reflects the time required for intermediate biological steps (such as transcription, translation, protein‐folding, nuclear import, or multisite phosphorylation) between the synthesis of the gene product and its repressive action. Conceptually, this delay plays a role analogous to the chain of intermediate reactions in the Goodwin model (Fig. 2A)—increasing the number of intermediate steps in the Goodwin model lengthens the effective delay between production and feedback, and in the limit of many steps, the chain can be approximated by a single discrete delay *τ*.

Despite its simplicity, this model captures essential features of TTFLs observed in circadian systems of *Neurospora*, *Drosophila*, and mammals. Extensions of this delay differential equation model have been studied in diverse biological contexts beyond circadian clocks [84], including somite segmentation oscillations [85, 86, 87] and cardiac rhythms [88], illustrating its broad applicability in modeling rhythmic phenomena. Numerical simulations of this system show that stable limit cycles emerge when the delay *τ* is sufficiently long and the nonlinearity sufficiently steep [77]. For example, circadian oscillations with a ~ 24‐h period can be generated with delays of approximately 6 h (corresponding to roughly one‐quarter of the period) while faster oscillators such as the human somite clock (with a ~ 5–6‐h period [89]) require shorter delays on the order of 1.5 h.

Oscillations only occur if the Hill coefficient *n* exceeds a critical threshold, corresponding to strong, switch‐like repression. As the nonlinearity increases, the system undergoes a Hopf bifurcation, and transitions from a steady state to a limit cycle regime. This bifurcation structure can be visualised by plotting oscillation amplitude as a function of *n*: for low *n*, the system converges to a stable fixed point, while above threshold, distinct peaks and troughs emerge corresponding to stable rhythms (similar to Fig. 2B).

Importantly, not all models represent delays explicitly as a parameter *τ*. Many ODE‐based TTFL models instead capture delays implicitly through chains of reactions, and the finite half‐lives of intermediate species, as in Fig. 2. In such models, the sum of these effective delays largely determines the period [90]. Experimental studies support this idea: altering the half‐lives of clock proteins (as in the familial advanced sleep phase syndrome FASPS [91]), changing nuclear import/export kinetics [92], or modifying phosphorylation timing [93, 94, 95] all result in corresponding shifts in the circadian period.

In *Drosophila*, for example, the nuclear import of the PER:TIM protein complex, which is part of the negative circadian feedback loop, introduces a delay of approximately 5.5 h [96]. In *Neurospora*, the FRQ protein (the negative regulator in the fungus' TTFL) undergoes extensive multisite phosphorylation on intrinsically disordered domains (with more than 100 phosphorylation sites) creating a slow feedback that underlies sustained oscillations [93, 97, 98]. Mammalian circadian clocks employ several distinct strategies to achieve similar effects: the stepwise assembly of large repressive protein complexes [99, 100], and epigenetic modulation of transcription and chromatin remodeling [101, 102, 103, 104, 105] have all been implicated in delayed feedback. Recent evidence also points to the role of biochemical timers: for instance, regulated slow ATPase activities are central to circadian timing in cyanobacteria [106, 107], and analogous mechanisms appear to modulate timing in eukaryotic systems as well [108]. These diverse and independently evolved solutions highlight a common functional requirement: introducing sufficient delay to stabilise and sustain circadian rhythms.

Interestingly, other mammalian TTFL‐based oscillators such as the segmentation (somite) clock [85, 90, 109], NF‐κB rhythms [110, 111, 112, 113], or p53 pulses [114, 115] exhibit much shorter periods, ranging from 2 to 5 h. In these cases, the required feedback delays are correspondingly shorter (on the order of 1 h), and could be explained by the time needed for transcription, translation, and protein modification alone. These differences reinforce the principle that the length of the feedback delay is a key determinant of oscillator period (Box 3), and that biological systems have evolved specific mechanisms that match delay timescales to their functional needs.

#### Wanted: nonlinearities!

Nonlinearities are equally crucial for sustaining limit cycle oscillations [69, 76, 81, 82] and stabilising clock amplitude. This means that any limit cycle oscillator model has nonlinear kinetic terms in its governing equations. In TTFLs, they often manifest as sharp, switch‐like ultrasensitive transitions that terminate transcription, achieved through mechanisms such as transcriptional repression by CRY and PER proteins, removal of activating complexes [116, 117], or epigenetic silencing *via* histone deacetylation [101, 118]. Other nonlinear sources include cooperative binding, molecular sequestration, and positive feedback loops (reviewed in [77, 119, 120, 121]), all of which create ultrasensitive responses commonly modeled by Hill kinetics. In the widely used Goodwin model, for instance, it has been shown that relatively large Hill coefficients are necessary to achieve limit cycle oscillations [42, 76, 122, 123, 124] (Fig. 2).

These nonlinear dynamics can produce bistability and hysteresis [77, 121, 125], phenomena observed in biological processes like cell cycle checkpoints [126, 127, 128, 129] and apoptotic signalling [130, 131]. Together, these nonlinear features stabilise oscillations and define the bounded, robust rhythmicity characteristic of biological limit cycles.

As a take‐home message from this section, it is important to highlight that rhythmic behaviour in biological systems is not simply a binary distinction between damped and self‐sustained oscillations. Instead, many systems operate in a ‘gray zone’, and rhythmic behaviour is not a fixed property of individual cells, but rather an emergent feature of their internal regulatory architecture and interaction with the environment. Whether a cell exhibits damped or self‐sustained oscillations depends on parameters such as coupling strength, temperature, metabolic state, and the architecture of transcriptional feedback. In practice, especially when analysing long, non‐stationary time series (as is often the case in circadian experiments) it can be challenging to distinguish clearly between damped and limit cycle dynamics [60]. Many cells may transition between these regimes depending on physiological context or experimental perturbations, highlighting the plasticity and context‐dependence of biological oscillations.

## *Come together right now*: synchronisation and coupling of biological oscillators

Biological rhythms rarely occur in isolation. From fireflies flashing in unison to SCN neurons harmonising circadian output, coordination is a hallmark of life. ‘Come together right now’ is a well‐known line from a Beatles song that captures the essence of synchronisation in biological systems; and Herzog and Aton [36] borrowed this iconic lyric as the title of a paper on circadian synchrony. Whether in the brain, liver or heart, the coordinated activity of many heterogeneous individual units gives rise to emergent system‐level rhythms. Such collective dynamics ensure robustness, despite noise and variability at the single‐cell level. This section explores the history, mechanisms and consequences of oscillator coupling in biological systems. We discuss the diverse patterns of synchrony observed in biological systems, highlighting how different modes of coupling and frequency relationships shape the temporal architecture of living organisms.

The scientific study of coupled oscillators traces back to the 17th century, when Christiaan Huygens observed two pendulum clocks mounted on the same beam gradually falling into synchrony, a phenomenon he described as ‘an odd kind of sympathy’ [8, 9]. In the 20th century, Van der Pol and Van der Mark investigated mutual synchronisation in nonlinear electrical circuits, motivated by cardiac pacemaker cells [132]. Around the same time, Erich von Holst introduced the concept of *relative coordination* to describe toroidal dynamics of coupled rhythmic movements of fish fins [133].

Mathematical theory advanced significantly in the second half of the 20th century. Arthur T. Winfree made key contributions to the study of biological oscillators, particularly in developing the theoretical framework for phase resetting [74]. He explored the role of phase response curves, phase transition curves and circle maps (see Box 4) in understanding how oscillators respond to perturbations. These tools became central to the mathematical analysis of synchronisation in biological systems. Yoshiki Kuramoto formalised a now‐classic model for large populations of weakly coupled phase oscillators [15], revealing how global synchrony can emerge from local interactions. Further conceptual advances were laid by Steven Strogatz, Michael Mackey, and Leon Glass [64, 65], who brought tools from nonlinear dynamics and bifurcation theory into physiology and circadian biology. Their work has made the theory of coupled oscillators a central framework for understanding biological timekeeping. Excellent reviews and textbooks [16, 17, 64, 65, 68, 75, 82, 134, 135, 136, 137, 138] summarise this body of work.

Box 4 **From phase response curves to the circle map**

Phase response curves (PRCs) describe how a brief stimulus shifts the phase of an oscillator, depending on when the stimulus occurs in its cycle. A powerful insight from nonlinear dynamics is that if stimuli repeat periodically and relaxation rates are high, the effect of the PRC can be generalised to an iterated map, known as the *phase transition curve* (PTC).

When the external stimulus is a periodic input, such as the daily light–dark cycle affecting a circadian clock, this iterated map becomes the circle map:

$$\varphi_{n+1}=\varphi_{n}+\Omega +K\sin\left(2{\pi \varphi }_{n}\right)\;\mathrm{mod}\;1.$$

Here, $\varphi_{n}$ is the oscillator's phase just before the *n*‐th stimulus; $\Omega =\left(\tau -T\right)/T$ quantifies the mismatch between intrinsic period *τ* and external period *T*; and *K* reflects the coupling strength. The sine term is a generic representation of the PRC's shape. The notation ‘mod 1’ means that after computing the update, only the fractional part is kept, ensuring that phase always lies within $\left[0,1\right)$; in other words, it wraps the phase back onto the unit circle.

This simple map captures essential features of entrainment: phase‐locking, phase dependence on intrinsic period and PRC shape, and transitions to complex behaviours like quasiperiodicity or chaos. Regions of stable synchronisation (e.g. 1 : 1 entrainment) appear as Arnold tongues, whose shape and size depend on Ω and K.

In circadian biology, this framework explains why individuals with longer intrinsic periods tend to entrain to later phases, and why synchronisation is still possible despite internal‐external mismatches. Importantly, the circle map builds a conceptual bridge from single‐pulse PRC measurements to full dynamical predictions under periodic forcing.

These concepts are nicely explained in the chapter by Granada et al. [68] and the great monograph by Glass and Mackey [65].

Coupling refers to the interactions among oscillators that influence their timing, either advancing or delaying their cycles. In circadian systems, coupling plays a critical role at multiple scales, from molecular feedback loops to cell–cell communication and organ‐organ coordination. Depending on the strength and nature of these interactions, and the degree of intrinsic period mismatch between oscillators, systems can exhibit a wide range of collective behaviours [43, 71], ranging from complete synchronisation to complex patterns such as amplitude modulations, multi‐frequency‐locking, and chaos.

Experimental evidence confirms that circadian clocks across tissues interact to maintain synchrony. The SCN remains the best‐studied example: even in slice cultures, SCN neurons retain robust synchrony through paracrine signalling and electrical coupling [36, 38, 40, 139, 140, 141, 142, 143, 144], highlighting the sufficiency of intercellular coupling for tissue‐level oscillations. Similar coupling mechanisms may operate among hepatocytes [41, 145, 146, 147]. Also in fibroblasts, paracrine [148, 149] and mechanical [150, 151] signalling may contribute to local coordination, though coupling among such cells is weaker and more variable. Because the nature and degree of coupling vary between tissues and conditions, quantifying the coupling strength and studying how such interactions modulate phase and period under physiological conditions remains a central challenge in chronobiology.

### Coupling strength and phase: keys to synchronisation

When two or more biological oscillators interact, their ability to synchronise depends on both, how strongly they influence each other, and when that influence occurs within each oscillator's cycle. These two features, *coupling strength* and *coupling phase*, jointly determine whether a population of oscillators will converge to a common rhythm or remain incoherent.

Coupling strength reflects how much the timing of one oscillator affects another's cycle. Even weak coupling can lead to stable phase relationships (phase‐locking) if the intrinsic periods of the oscillators are similar [43]. As coupling becomes stronger, synchronisation can be achieved even in the presence of moderate period mismatches [43]. However, when the difference in natural frequencies exceeds a critical threshold, oscillators may fail to synchronise, producing drifting phases, amplitude beats, or complex quasiperiodic behaviour [152].

Just as important as the strength of coupling is its timing, known as the phase of coupling [153, 154, 155, 156]. For example, in systems where oscillators influence each other with a time delay (e.g. via neuropeptide release or transcriptional feedback [153]), the relative phase of interaction can qualitatively change the system's behaviour. A well‐timed signal may promote synchrony, while a poorly timed one may destabilise it or generate phase waves [157].

Theoretical models help formalise these intuitions. The Adler Equation [158], Kuramoto model [15, 157], and circle maps (see Box 4) describe how phase differences evolve over time under various coupling regimes. Central to these analyses are phase response curves [65, 68, 74], which characterise how a perturbation shifts an oscillator's phase depending on when it occurs in the cycle. In discrete systems, phase transition curves [65, 68, 74] serve a similar role. These tools allow researchers to predict whether oscillators will synchronise to each other, or to an external zeitgeber such as the light–dark cycle.

**Fig. 3:**
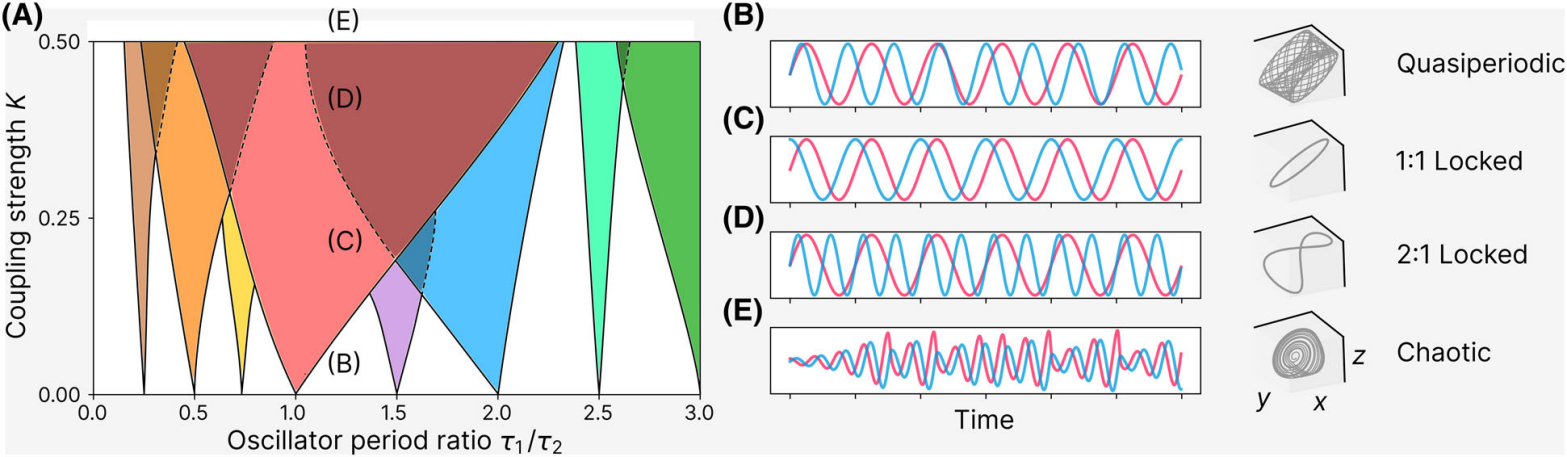
Dynamical regimes of two coupled oscillators as a function of coupling strength and period ratio. (A) Arnold tongue diagram showing regions of different dynamics as a function of oscillator period ratio $\left(\frac{\tau_{1}}{\tau_{2}}\right)$ and coupling strength *K*. (B–E) Representative time series (left) and corresponding phase portraits (right) illustrating the main dynamical regimes of the two oscillators at selected points in (A), as the coupling strength *K* increases along the vertical axis: (B) quasi‐periodic dynamics, (C) 1 : 1 phase‐locking, (D) 2 : 1 locking, and (E) chaotic dynamics at very high coupling. In (D), the 1 : 1 and 2 : 1 tongues overlap, meaning that both locking regimes can coexist in this region. However, only the 2 : 1 locked dynamics are shown here for clarity. Chaotic dynamics are shown outside the 1 : 1 tongue, where both oscillators exhibit aperiodic behaviour and do not lock in an *n* : *m* fashion.

Graphical methods such as *Arnold tongue* diagrams [65, 159] visualise the regions in parameter space where synchronisation occurs. These ‘tongues’ emerge in plots of coupling strength versus frequency mismatch, showing that stronger coupling widens the range of periods over which synchronisation is possible (Fig. 3A). These concepts are central to understanding how circadian cells entrain to environmental cues, or how SCN neurons achieve internal coherence despite variability in their intrinsic periods.

### Types of synchronisation observed in biology

Biological systems exhibit a wide variety of synchronisation behaviours, shaped by the strength of coupling, intrinsic period mismatches, and system nonlinearities. Below, we outline several synchronisation regimes observed in biological systems, arranged from more common to more complex forms (Fig. 3B–E).

#### 1 : 1 Frequency‐locking: ubiquituous in circadian biology

A widespread form of synchrony among biological oscillators is 1 : 1 frequency‐locking, in which coupled oscillators align their periods and maintain a stable phase relationship (Fig. 3C). This mode of coordination is seen in systems as diverse as the left and right vocal folds, which oscillate in phase to produce a steady tone during healthy voice, and circadian networks, where synchronisation among cells generates coherent daily rhythms. In the SCN, for instance, individual neurons with heterogeneous intrinsic periods synchronise via gap junctions and neurotransmitter signalling, such as VIP and GABA, to produce a unified tissue‐level oscillation [36, 38, 40, 139, 140, 141, 142, 143, 144]. Peripheral tissues such as the liver [41, 145, 146, 147], skin [148, 149], and lung [37] exhibit similar locking [3, 4, 160] despite distinct tissues having different phases [161, 162, 163].

Importantly, frequency‐locking does not imply that all oscillators share the same phase. In the SCN, for example, stable phase waves span its dorsoventral axis [164, 165, 166, 167, 168]. The hallmark of 1 : 1 locking is not perfect synchrony, but stable phase differences that persist over time [43]. A similar phenomenon occurs in vocal physiology: the left and right vocal folds maintain phase‐locked oscillations to produce a stable tone in a healthy voice [169].

#### *n* : *m* synchronisation: rhythms in ratio

In some systems, oscillators settle into *n* : *m* frequency‐locking, where one completes *n* cycles for every round of *m* cycles of the other (Fig. 3D). This is often observed when oscillators with harmonically related periods interact [65, 133]. In marine organisms, multiple endogenous clocks (including circadian, tidal and lunar) can coexist and interact [170, 171, 172]. While 12.4‐h tidal rhythms are typically entrained to the tides and may drift relative to the light–dark cycle, recent research suggests that coupling between tidal and circadian clocks is possible [170], and under certain conditions, transient or partial 1 : 2 locking might emerge, especially when environmental cues (such as light and pressure) align.

In mammals and fungi, 12‐h rhythms have been identified at the transcriptomic and proteomic levels [171, 173, 174, 175, 176]. However, their mechanistic origin remains debated, with alternative hypotheses involving independent ultradian clocks [172, 177]. Distinguishing between these scenarios will require further research.

Beyond molecular rhythms, physiological oscillators such as respiration and heartbeat can also exhibit coupling. For instance, the relationship between breathing and cardiac cycles has been interpreted as a form of phase‐locking, typically following an approximate 1 : 4 or 1 : 5 ratio depending on the organism and physiological state [65, 178]. These interactions illustrate how *n* : *m* synchronisation can arise across multiple biological scales, from gene expression dynamics to whole‐organism physiology.

#### Relative coordination and quasiperiodicity: dancing without locking

When coupling is weak or period mismatches are too large, oscillators may fail to lock, yet still exhibit structured but non‐repeating patterns. This state, termed relative coordination [133] or quasi‐periodicity, arises when the ratio of their periods $\frac{\tau_{1}}{\tau_{2}}$ is not a rational number $\frac{n}{m}$. Instead of converging to a stable phase relationship, the system exhibits a non‐repeating, yet organised pattern. In phase space, such dynamics correspond to motion on a torus (a doughnut‐shaped surface, Fig. 3B), with slow phase drift and amplitude modulations.

An interesting experimental example comes from rodents subjected to unusual light cycles. Under certain lighting regimes, the circadian locomotor rhythm splits into two independent activity bouts, possibly reflecting desynchronised SCN neuron clusters [179, 180, 181, 182].

#### Multistability and hysteresis: rhythmic regime shifts

Coupled oscillators can support multiple coexisting rhythmic states (overlapping regions in Fig. 3A), a phenomenon known as birhythmicity [61, 62]. In these multistable regimes, the same system may exhibit distinct phase relationships or wave patterns under identical parameter values, depending on initial conditions or past states. Transitions between these states can be triggered by gradual parameter changes such as neuropeptide signalling, light or temperature. Importantly, when such transitions occur through gradual variation of parameters, the system often exhibits hysteresis: the switching point depends on the direction and history of the parameter change, so returning to a previous state requires reversing the change beyond the original switching threshold [77, 125]. This hysteresis provides a form of memory that stabilises rhythmic states against transient fluctuations.

This behaviour is well documented in vocal fold oscillations, where a small change in airflow or tension can cause an abrupt switch between vocal registers (chest to falsetto) [183, 184, 185, 186, 187]. Similar transitions are postulated in circadian networks during jet lag or seasonal adaptation [188, 189, 190, 191].

#### Period‐doubling and chaos: into the wild

Finally, under conditions of strong nonlinear coupling, systems may undergo period‐doubling bifurcations and ultimately display deterministic chaos (Fig. 3E) [65, 66, 152, 192]. Chaotic dynamics are aperiodic and exquisitely sensitive to initial conditions [193]. Though well studied in theory [64, 65], biological evidence for chaos in circadian systems is limited [173, 182], due to the long timescales that are needed to characterise such dynamics, as well as inherent noise in biological measurements. However, simulations suggest that chaotic dynamics could emerge under specific parameter regimes [61, 62, 73, 192], potentially playing a role in extreme physiological or pathological conditions. Interestingly, chaotic dynamics have been described in vocal systems, for instance, in infant cries [194] and animal vocalisations of tigers [195] or crocodiles [196], suggesting that biological oscillators can traverse both periodic and aperiodic rhythmic regimes [152].

The interplay of delays, nonlinearities and coupling gives rise to a rich repertoire of dynamical behaviours, from robust phase‐locking to quasi‐periodicity and chaos, that mirrors the diversity of biological rhythms across systems. Tools from nonlinear dynamics offer a predictive lens into how biological timing systems function, adapt and fail. Yet despite these advances, quantifying coupling strength in real biological systems remains a major challenge. Coupling is not always direct or symmetric [15, 197], and it may involve multiple modes of interaction (chemical, electrical, mechanical), often with time‐varying or state‐dependent properties. Moreover, the effects of coupling depend critically not just on its strength, but on the precise timing of interactions relative to intrinsic cycles [153]. Disentangling these elements experimentally is difficult but essential for connecting models to data, and for understanding how synchrony is maintained or lost in development, disease, or across environmental contexts.

## *Here comes the sun*: Entrainment of circadian clocks to zeitgebers

Living organisms are immersed in rhythmic environments. The most pervasive of these is the 24‐h light–dark cycle, which shapes behaviour, physiology, and gene expression across species. To function optimally, internal circadian clocks must align with these external cues, a process known as entrainment [5, 6, 198, 199]. Unlike mutual synchronisation between coupled clocks, entrainment reflects a form of unidirectional coupling: an external oscillator, called the zeitgeber (German for ‘time‐giver’), influences the phase and period of the internal clock without itself being perturbed. Among these zeitgebers, light stands out as the most potent zeitgeber, resetting our biological clock every day. But other signals, such as temperature cycles, feeding schedules and even social routines, can also serve as powerful zeitgebers [200], particularly in peripheral tissues.

‘Here comes the sun’, sang The Beatles, and with it, the daily resynchronisation of physiology with the external world. This capacity to lock onto environmental rhythms underlies biological adaptation to day and night, seasons, and social routines.

### Defining entrainment and shaping the phase of entrainment

Entrainment refers to the process by which internal circadian rhythms synchronise to an external periodic signal, or zeitgeber. From an evolutionary perspective, entrainment is not just a mechanistic property of clocks, but it is the primary target of selection [5, 6, 201]. What matters most for fitness is not the intrinsic period of the clock per se, but rather the phase of entrainment (*ψ*, that is, the stable timing relationship between the internal rhythm and environmental cycles) [202]. This phase relationship determines when key physiological processes, such as sleep, feeding, and hormone secretion occur relative to the zeitgeber cycles. For example, individuals with familial advanced sleep phase syndrome (FASPS) or strong morning chronotypes tend to exhibit much earlier phases of entrainment [58, 91, 203]. In both animal and human studies, *ψ* is often estimated from proxies such as activity onsets [5, 204] or mid‐sleep times [58].

Two important aspects of entrainment are the entrainment range, which defines the span of zeitgeber periods for which stable synchronisation occurs, and the phase of entrainment (*ψ*), which captures the precise temporal alignment of the internal clock to the external signal. While clocks may adopt the same period under 1 : 1 locking, they do not necessarily align in phase. Indeed, stable inter‐individual differences in *ψ* (the basis of chronotypes, such as ‘morning larks’ and ‘night owls’) arise even under identical zeitgeber schedules [58, 198, 202, 205, 206, 207, 208]. These differences reflect variation in intrinsic period, amplitude, and the capacity to shift in response to perturbations.

Although the oscillator's intrinsic period influences the phase of entrainment (shorter‐period clocks tend to lock to an earlier *ψ*, and longer‐period clocks to a later one) [55, 56], *ψ* is shaped by more than period alone. Other internal properties, such as amplitude, also play a critical role: low‐amplitude oscillators are more easily shifted and exhibit broader entrainment ranges [37, 188, 209]. Features like twist (the co‐variation of period and amplitude during transients, see Box 2) [73] and waveform shape [210] influence how clocks settle into their entrained state after shifts or perturbations. Additionally, seasonal changes in photo‐ or thermoperiod modulate the entrainment phase [211, 212], by altering the timing and duration of the zeitgeber exposure. The entrainment phase, therefore, emerges from a dynamic interplay between intrinsic oscillator dynamics and external cues.

Real‐world studies provide compelling illustrations: in outdoor camping experiments without artificial light, individuals consistently shift toward earlier phases of entrainment [213, 214], a phenomenon that aligns with predictions from oscillator models [56, 81]. Similar effects are seen in rural Brazilian and Argentinian populations with limited access to electric lighting [215, 216]. These findings underscore the plasticity of circadian phase and show that entrained timing is as much a function of environmental context as it is of individual biology.

### The geometry of entrainment: theoretical tools for understanding entrainment

To understand how circadian clocks synchronise with external rhythms, oscillator theory provides a set of geometric and dynamical tools that describe how phase and amplitude respond to periodic inputs. These tools help explain not only whether entrainment occurs, but also how robust it is, how quickly it is achieved, and how the phase of entrainment depends on internal and external parameters.

#### Phase response curves and Arnold tongues

One of the most widely used tools is the *phase response curve* (PRC), which characterises how a stimulus shifts the oscillator's phase depending on when it occurs in the cycle [65, 68, 217]. For example, a light pulse at the beginning of a subjective night may delay the clock of a diurnal animal, while the same pulse near the end of the night may advance it. PRCs can be measured experimentally and serve as a ‘fingerprint’ of the oscillator, predicting how easily it entrains, how it responds to different zeitgeber periods, and whether it exhibits abrupt phase shifts [218, 219].

**Fig. 4:**
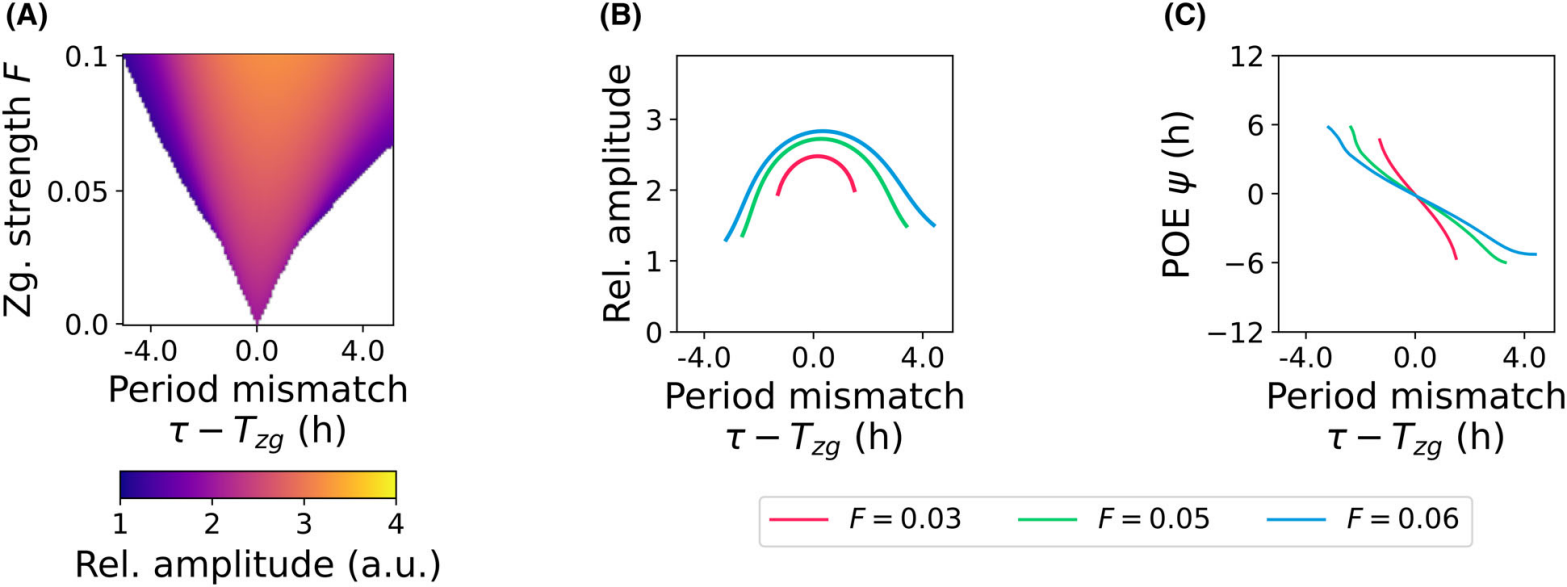
Theoretical representations of circadian entrainment. (A) Arnold tongue diagram of a Poincaré oscillator (Box 2) driven by a periodic zeitgeber, illustrating the 1 : 1 entrainment region as a function of zeitgeber strength *F* and period mismatch between the intrinsic oscillator *τ* and the external zeitgeber *T*_*zg*_. Stronger zeitgebers expand the range of periods over which stable entrainment occurs. (B) Resonance curves showing how increasing zeitgeber strength *F* leads to larger amplitude responses of the oscillator. The largest amplitude expansion occurs when the zeitgeber period matches the intrinsic oscillator period, consistent with resonance phenomena described in the manuscript. (C) Phase of entrainment (POE) as a function of period mismatch, illustrating the 180° rule [55, 56]. Across the entrainment range, small changes in the period ratio can lead to large phase shifts, particularly for narrow entrainment ranges.

Another key representation is the *Arnold tongue diagram* (as previously discussed), which maps regions of stable frequency‐locking as a function of zeitgeber amplitude and period mismatch (Fig. 4A). The triangular ‘tongue’ emerging from the 1 : 1 locking condition shows that stronger inputs expand the entrainment range. Similar diagrams describe higher‐order locking (e.g. 2 : 1, 3 : 2), where the oscillator completes multiple cycles per zeitgeber cycle.

#### Resonance revisited: amplitude expansion in entrained oscillators

As introduced previously, resonance describes the amplification of an oscillator's response when the driving period *T* closely matches its intrinsic period *τ*. In the context of entrainment, this manifests as an increase in oscillation amplitude near the centre of the 1 : 1 locking region (Fig. 4B). While this resonance‐like behaviour is a hallmark of damped oscillators, it also appears in forced limit‐cycle systems, where the shape and steepness of the resonance curve depend on both the zeitgeber and the internal oscillator properties.

From the external side, stronger zeitgebers (i.e. larger forcing amplitudes) generate steeper and more pronounced resonance curves, broadening the region of enhanced amplitude and expanding the entrainment range (Fig. 4B). Conversely, weak forcing produces shallower amplitude modulation and narrower resonance zones. Internally, the amplitude relaxation rate (i.e. the rate at which perturbations are attracted back to the limit cycle, see Box 2) plays an equally critical role: oscillators with high relaxation rates (strongly attracting cycles) exhibit flatter, less pronounced resonance curves, whereas those with slow relaxation display sharper amplitude amplification near frequency‐matching [37]. Thus, the prominence of resonance reflects a dynamic interplay between external drive and intrinsic damping, integrating both sides of the oscillator‐zeitgeber relationship.

Biologically, such amplification has been observed in cyanobacteria, where strains with intrinsic periods closely aligned with the environmental cycle exhibit enhanced rhythmic amplitude and outcompete others [220, 221]. This highlights how resonance shapes not only entrainment dynamics but also evolutionary outcomes, favouring clocks tuned to their environmental zeitgeber periods.

#### Phase of entrainment and the 180° rule

Another key feature of entrainment is the variation in the phase of entrainment *ψ* within the 1 :1 locking region. In theoretical models, *ψ* can shift by up to 180°, depending on the mismatch between *τ* and *T* (Figs 1C and 4C). This property, known as the 180° rule [55, 56], implies that even small differences in intrinsic period can lead to large shifts in the timing of entrained rhythms. This steep phase dependence is especially pronounced near resonance, where small changes in *T* can cause rapid shifts in *ψ*.

Experimental studies in human chronobiology support this prediction. For example, a modest 12‐min difference in intrinsic circadian period has been shown to produce a 90‐min shift in activity phase [22, 58, 205, 206, 207, 208]. This extreme sensitivity helps explain the diversity of chronotypes in the population, where individuals with slightly longer or shorter intrinsic periods tend to entrain to later or earlier phases, respectively.

#### Beyond PRCs: surfaces and maps

While PRCs and Arnold tongues provide powerful insights, more recent approaches offer richer representations of entrainment. One such framework is the *circadian surface* [202, 212, 222], which maps how the phase of entrainment depends on the interplay between the internal period, the zeitgeber period and photoperiod. These empirically derived surfaces reveal systematic and often nonlinear dependencies that challenge traditional PRC‐based models.

Complementary to these empirical mappings are *entrainment maps* [223], which offer a mechanistic, model‐based approach. By iterating a one‐dimensional phase map derived from the oscillator's dynamics under periodic forcing, these maps can predict not only the phase of entrainment for any photoperiod, but also the stability of the entrained state and the time required to achieve it.

Together, these theoretical tools define the geometry of entrainment in precise dynamical terms. Phase response curves describe how periodic inputs shift the oscillator's timing, Arnold tongues map the parameter ranges that support stable frequency‐locking, and resonance curves capture how amplitude responds to the match between internal and external periods. In combination with circadian surfaces and entrainment maps, these approaches provide a quantitative framework for predicting when and how entrainment occurs, how robust it is to perturbations, and how phase and amplitude relationships arise from the interaction between the clock and its zeitgeber.

### What makes a clock easy to reset? Weak versus strong oscillators

Circadian oscillators differ in how easily they adjust to external perturbations and timing cues. This difference is often framed in terms of weak versus strong oscillators; a distinction rooted in classical oscillator theory [37, 55, 56, 65], but highly relevant to biological systems. In this framework, strong oscillators are characterised by their robustness: they maintain stable, self‐sustained rhythms and resist phase resetting, entraining only within narrow parameter ranges. Weak oscillators, by contrast, are more plastic: they adjust easily to changing zeitgebers and exhibit broad entrainment ranges, but are also more sensitive to noise and perturbations.

#### Multidimensional determinants of oscillator strength

Oscillator strength is not defined by a single parameter, but rather emerges from the interplay of several dynamical properties. Among the most relevant are the amplitude of the rhythm, the amplitude relaxation rate, and the degree of intercellular coupling. While these dimensions often co‐vary, they are not strictly correlated and can be independently tuned in theoretical models and biological systems.

The amplitude reflects the size of the limit cycle and is often associated with the robustness of the rhythm. However, amplitude alone does not determine how quickly an oscillator returns to its steady‐state trajectory after a perturbation. This recovery speed is captured by the amplitude relaxation rate, which quantifies how rapidly deviations from the limit cycle decay (see Box 2). For example, in the Poincaré oscillator, the amplitude remains fixed while the relaxation rate parameter *α* can be varied independently, illustrating that these two quantities are dynamically distinct. A third axis is coupling, particularly relevant in multicellular systems. Strong intercellular coupling can enhance both the effective amplitude and the relaxation rate of the collective rhythm, but this is not always the case. In the SCN, for instance, robust coupling ensures high phase coherence and resistance to perturbation, even though the amplitude of individual gene expression rhythms has been shown to be modest [162, 224].

Together, these parameters define a multidimensional space in which oscillator strength resides. Rather than being reduced to a single number, strength reflects the overall ability of a system to maintain rhythmicity, resist perturbations, and entrain selectively to external signals.

#### Manifestation in biological systems

The multidimensional nature of oscillator strength is reflected in diverse biological systems. Weak oscillators, such as those found in unicellular organisms or peripheral tissues, often exhibit low amplitude and large PRCs. These features make them highly sensitive to external cues: even small stimuli can induce substantial phase shifts, enabling broad entrainment across a wide range of zeitgeber periods and waveforms [57, 202, 225]. For instance, peripheral clocks can be rapidly reset by hormonal signals [59], feeding schedules [145], or temperature cycles [37, 226], even when the central clock remains largely unaffected [227].

At the single‐cell level, circadian rhythms often display reduced amplitude and can be modeled even as damped oscillators [60]. Whether these rhythms persist or decay depends on both intrinsic oscillator properties and the cellular environment. At the tissue level, intercellular coupling becomes a key determinant of collective behaviour. In the SCN, strong coupling among neurons supports coherent, self‐sustained rhythms even in vitro [37, 38, 139]. In contrast, peripheral tissues such as the lung or fibroblasts exhibit weaker coupling, leading to desynchronisation and apparent damping [228]. The liver represents an intermediate case: it is one of the most robust peripheral oscillators [229], yet still loses coherence when isolated from SCN input [145].

#### A continuum between robustness and flexibility

The distinction between weak and strong oscillators reflects a fundamental trade‐off between robustness and flexibility. Strong oscillators offer temporal stability and resistance to noise but are less adaptable to changing environments, whereas weak oscillators are easily reset and entrained but more susceptible to disruption. In this review, we use the terms *strong* and *weak* in a broad, functional sense to describe an oscillator's entrainability and sensitivity to zeitgebers, rather than as labels tied to a single parameter. Strong oscillators are characterised by narrow entrainment ranges and small phase response curves (PRCs), while weak oscillators exhibit broader entrainment and larger PRCs [22, 37, 205, 230, 231].

In multicellular organisms, both types coexist and interact. Peripheral clocks, often weak in isolation, can rapidly adjust to feeding or hormonal cues, while the SCN acts as a strong, stable pacemaker anchored to the light–dark cycle. System‐level entrainment emerges from this interplay, with oscillator strength shaped by the combined effects of amplitude, relaxation rate, and coupling. Ultimately, oscillator strength is best viewed as a multidimensional and context‐dependent property, capturing how intrinsic dynamics and network interactions jointly determine an oscillator's ability to maintain rhythmicity and respond to environmental cues.

### Chronotherapy: harnessing oscillator‐coupling for cancer treatment

Having examined the biological and physical mechanisms shaping circadian regulation, we now turn to a therapeutic paradigm that translates these principles into medical practice: chronotherapy. This approach recognises that the *when* of treatment can be as critical as the *what* or *how much*, making time itself an important variable in the therapy of cancer and other diseases. Francis Lévi pioneered chronotherapy in oncology by demonstrating that cytotoxic chemotherapy scheduled according to circadian rhythms can significantly improve patient outcomes. In a landmark trial in the mid 90s, colorectal cancer patients receiving chronomodulated 5‐fluorouracil (5‐FU), leucovorin and oxaliplatin achieved higher response rates (53% versus 32%) and far fewer severe side effects compared to constant‐rate infusion [232]. These findings underscore the principle of temporal sensitivity, where aligning drug delivery with circadian rhythms can simultaneously enhance efficacy and reduce toxicity. Recent editorials have called for integrating circadian medicine into routine clinical practice, outlining three fundamental aspects: detecting, targeting and exploiting the clock to optimise patient care [233].

#### From cellular oscillators to tumour networks

Chronotherapy can be conceptualised through the lens of coupled oscillator theory. Rather than one clock acting in isolation, the body comprises interacting oscillators: the central circadian pacemaker, peripheral tissue clocks, the cell cycle, and immune cycles [234]. Their interplay depends on their intrinsic period, coupling strength, and phase relationship. Here, strong coupling can enforce synchrony (e.g. daily alignment of the cell cycle with circadian time), while weak coupling or mismatched frequencies may produce irregular rhythms. This framework helps explain why treatment given at one phase can be protective, while at another phase it can even be harmful [235].

Bieler et al. showed that mammalian fibroblasts maintain a robust 1 : 1 mode‐locked relationship between circadian and cell‐cycle oscillations, with cell division occurring at a consistent phase of the circadian cycle [236]. In parallel, Feillet et al. reported multiple phase‐locking modes between the two oscillators, including 1 : 1 and higher‐order relationships, and demonstrated that cell division can reset circadian phase, indicating bidirectional coupling [237]. Together, these studies revealed stable and versatile coupling mechanisms linking circadian and cell‐cycle clocks. Extending these insights to cancer, Gérard and Goldbeter showed that strong circadian coupling can lock cancer cell division cycles to a 24‐h rhythm, while weak coupling leads to 48‐h or chaotic dynamics [238]. Building on these ideas, Gutu demonstrated experimentally that circadian and cell‐cycle clocks form a dual‐oscillator system in tumour cells [239]. When circadian coupling is intact, coherent 24‐h rhythms emerge at the population level across cancer cells, driving oscillatory growth patterns. Disrupting this coupling, through clock gene knockout or blocking synchronising signals, such as TGF‐β, desynchronises cells and leads to irregular proliferation [239].

These oscillator dynamics extend to the tumour‐host interface. Scheiermann and colleagues revealed that tumour‐infiltrating CD8^+^ T cells follow daily rhythms shaped by two interacting clocks: the intrinsic circadian machinery of the T cells themselves and the rhythmic expression of adhesion molecules regulated by endothelial clocks in tumour blood vessels [240]. They further demonstrated that circadian timing can modulate the efficacy of both CAR T‐cell therapy and anti‐PD‐1 immune checkpoint blockade, underscoring the importance of synchronising treatment timing with the rhythmic immune milieu. In addition, Herzog and colleagues showed that glioblastoma cells synchronise with host circadian rhythms via glucocorticoid‐signalling, creating a coupled tumour‐host system that accelerates growth when synchronised, and slows growth when disrupted [241]. The tumour microenvironment thus operates as a dynamic network where immune infiltration, tumour growth, and drug sensitivity oscillate in coordinated rhythms.

#### Circadian heterogeneity and personalised timing

Despite these theoretical and experimental advances, circadian properties in cancer are far from uniform. In a recent study, 14 breast cancer cell lines were profiled, revealing four broad circadian phenotypes: cells with robust clocks, cells with weak or unstable oscillations, and cells with an effectively dysfunctional circadian clock [242]. This heterogeneity, observed even within a single cancer type, poses a major challenge for clinical translation and underscores the need for personalised chronotherapeutic strategies.

Adding to this complexity, cancer patients are exposed to multiple external time cues. While the light–dark cycle dominates central clock entrainment, feeding schedules, individual sleep–wake patterns, and drug administration times can all reset peripheral clocks. Recent findings demonstrated that zeitgebers can reinforce rhythmicity when aligned in phase, but compete or cancel out when misaligned [154]. This highlights an important principle: therapeutic timing must not work against the body's other rhythms but ideally integrate with them [154].

#### Therapeutic frontiers of chronotherapy

Chronotherapy has progressed from early proof‐of‐concept in chemotherapy to a broader framework that has the potential to span multiple cancer treatment modalities. By aligning therapy with circadian biology, clinicians can exploit temporal windows where tumours are most vulnerable and healthy tissues most resilient.

Lévi's protocols demonstrated how tailoring drug infusion to circadian phases can increase tolerability and efficacy [232]. Mathematical models have since enabled individualised schedules by incorporating patient circadian markers and pharmacokinetic profiles [243, 244, 245]. Meanwhile, preclinical high‐throughput screens enable systematic evaluation of diverse cell models and drugs in a time‐of‐day‐dependent manner, allowing researchers to rank treatments by their timing benefit through ‘chronotherapeutic indices’ [246].

CAR T‐cell therapy, a highly personalised anti‐cancer treatment where genetically modified T‐cells directly target and kill cancer cells, represents a new frontier for chronotherapy applications. Here, the cyclic nature of immune function has been shown to create distinct windows of enhanced tumour control and lowered toxicity in mouse models [240], with early clinical data beginning to support similar time‐of‐day effects in patients [247].

Together, these advances demonstrate that time can be harnessed as a therapeutic variable across diverse treatment strategies, positioning chronotherapy as a cornerstone of future precision oncology.

## Discussion: unifying principles and open questions

In this review, we have argued that circadian clocks are best understood not as isolated timers but as interacting dynamical systems. This oscillator‐based view helps explain how rhythms are generated, sustained, and coordinated across different biological contexts. It also highlights the underlying principles of delayed feedback, nonlinearity, coupling and entrainment, that govern both the stability and plasticity of circadian behaviour. Several themes emerge from this perspective, pointing to conceptual bridges between molecular mechanisms and systems‐level dynamics.

### How rhythms arise from delayed feedback and nonlinearities

At the heart of circadian timekeeping are limit cycles: self‐sustained oscillations that persist after transient disturbances and return to a stable periodic trajectory (Fig. 2). As discussed previously, such dynamics arise when delayed negative feedback and sufficient nonlinearity, together with a continuous supply of metabolic energy, push the system beyond a Hopf bifurcation. Sustained rhythms require an ongoing input of free energy, for instance, ATP‐dependent transcription, translation, protein turnover, and post‐translational modifications continuously drive the molecular clockwork. These ingredients reflect fundamental features of circadian architecture: delays emerge from slow processes such as transcription, translation, and protein modification [97, 248], while nonlinearities act as network switches [77, 119, 120, 121] and arise from cooperative binding, sequestration, and chromatin remodelling. Together, they shape both the period and the amplitude of the rhythm, with the limit cycle period being 3–4 times the delay. Interestingly, some circadian systems [60], as well as other cellular oscillators such as p53 dynamics and the somite segmentation clock, appear to operate close to the edge of rhythmicity, suggesting that transitions between damped and self‐sustained regimes may occur dynamically, depending on context. This view emphasises the flexibility of biological clocks and highlights the role of model parameters and thus the cellular environment in shaping rhythmic behaviour.

### Keeping the band together

Second, coupling introduces collective behaviour. From the perspective of oscillator theory, what makes a population of clocks coherent is not just that they tick at the same speed, but that they interact: shifting each other's phases, reinforcing synchrony, or introducing complexity. In the SCN, for instance, coupling through neuropeptides such as VIP, gap junctions, and synaptic connections [139, 140, 141, 142, 143, 144] enables coherent population‐level rhythms despite intrinsic variability across neurons. Theoretical models, from phase oscillator approximations [16, 157] to network simulations [43, 44, 249], have shown that coupling strength critically shapes collective dynamics: stronger coupling narrows the distribution of intrinsic periods (i.e. results in frequency pulling), reduces phase dispersion, and can amplify rhythmic amplitudes [43]. These effects are not limited to the SCN; similar principles apply in somite clocks [156], pacemaker cells in the heart [65], or flashing fireflies [250].

From a dynamical systems perspective, coupling is characterised not only by its strength but also by its timing. Coupling delays in paracrine signalling, for example, can qualitatively change synchronisation outcomes [153, 156]: sometimes enhancing stability, but other times promoting desynchronisation. Such effects have been linked to age‐related changes in SCN coherence [251, 252], to altered phenotypes in *Cry*‐deficient mice [253], and to the SCN's adaptation to seasonal changes [188, 190]. Simulations suggest that the interplay of coupling strength and coupling delay defines the architecture of synchrony, but experimentally, both remain difficult to quantify. Monitoring phase distributions or period variance has been proposed as a proxy for coupling strength [41, 43], but robust experimental methods to quantify coupling in vivo are still lacking, especially in heterogeneous, dynamic tissues. As a result, despite the wealth of theoretical tools, connecting model parameters to biological measurements remains an open challenge.

### Entrainment, chronotypes and chronotherapy

A third key feature of circadian systems is their capacity to entrain to external zeitgebers such as light, feeding, or temperature cycles. Oscillator theory provides a powerful framework to understand this alignment. Core quantities include the phase of entrainment (the stable timing relationship between internal and external rhythms) and the entrainment range (the span of zeitgeber periods over which stable locking occurs). These properties are shaped by oscillator amplitude, intrinsic period, waveform, as well as strength and period mismatch of the input signal.

Graphical tools such as Arnold tongue diagrams (Figs 3 and 4) illustrate how synchronisation emerges and breaks down. These diagrams plot entrainment regions in parameter space and reveal how small changes in the zeitgeber period can induce amplitude changes due to resonance or abrupt shifts in the phase of entrainment. In systems with narrow tongues, even slight period mismatches lead to large phase differences, providing a theoretical basis for chronotype diversity. Extensions of this formalism, such as Arnold onions [209, 211], further incorporate photoperiod length and have been used to model seasonal adaptation and entrainment in high‐latitude environments [191, 254, 255].

These geometric insights are not just mathematical abstractions: they help explain entrainment dynamics in both laboratory and real‐world settings, including rapid phase shifts during travel or shifts in sleep timing in response to artificial light. They also inform translational strategies. Chronotherapy represents a systems‐level intervention in which therapeutic success is shaped not only by the molecular identity of a drug but also by the temporal structure of the biological processes it targets. Cell proliferation, DNA repair, immune surveillance, and drug metabolism all follow circadian rhythms, creating windows of vulnerability and resilience that can be exploited. By synchronising internal oscillators with external zeitgebers and aligning treatment schedules accordingly, therapy can be delivered when tumours are most sensitive and healthy tissues least affected.

This perspective reframes cancer not only as a genetic and molecular disease but also as a disorder of time. It emphasises that circadian disruption, whether intrinsic to tumour cells or imposed by external schedules, can profoundly alter treatment outcomes. Addressing this requires integrating oscillator theory, experimental profiling of circadian phenotypes, and patient‐specific timing cues into therapeutic design.

The central lesson is that timing is not a passive background condition but an exploitable dimension of therapy. Used with precision, it offers a means to shift the balance in favour of protecting healthy tissues while targeting cancer more effectively, making chronotherapy an essential part of future precision oncology.

### Open questions and broader implications

Finally, an important challenge lies in integrating rhythms across different temporal scales. Circadian clocks do not operate in isolation: they interact with ultradian rhythms (those with periods shorter than 24 h) [171, 174, 175, 176, 177, 256], and with infradian rhythms (with periods longer than 24 h, such as lunar or menstrual cycles [257, 258]). They are also strongly influenced by stochastic components [72, 259, 260, 261]. While many tools in nonlinear dynamics (e.g. phase response theory, Arnold tongues, circle maps) assume quasi‐deterministic behaviour, biological systems are noisy, plastic, and evolving. This raises important questions: To what extent can oscillator theory capture such variability, including transient or weakly‐expressed rhythms that may nonetheless shape system behaviour [262, 263]? And where do we need new tools or concepts to interpret rhythms that are irregular, transient, or highly heterogeneous?

Addressing these questions will require combining theoretical and experimental approaches, and finding a balance between abstraction and biological mechanistic detail. The framework of oscillator theory remains a valuable starting point, but it must be extended and adapted to reflect the complexity of real systems. Doing so may help us better understand not just how rhythms are generated, but how they respond, adapt, and sometimes fail under changing physiological or environmental conditions.

## Conclusions: coupled oscillators as a framework for circadian systems

Oscillator theory has provided valuable insights into circadian systems, clarifying how delayed feedback, nonlinearity, and coupling give rise to self‐sustained rhythms, and how these rhythms synchronise with environmental cues. It offers a framework that connects molecular feedback loops with emergent behaviours at the tissue and organismal level. Yet, many open questions remain. How is coupling regulated in complex tissues, such as the SCN or pancreatic islets? What defines whether an oscillator behaves as weak or strong, and how do these properties shift in ageing or disease? And as new clocks with distinct periods and entrainment properties are identified across tissues, how do these heterogeneous oscillators coordinate to maintain organism‐wide temporal coherence?

More broadly, the principles reviewed here extend well beyond circadian biology. Limit cycles, synchronisation, and entrainment are relevant in systems ranging from cardiac pacemakers and vocal fold dynamics to somite segmentation and stress‐response networks, such as NF‐κB or p53. As experimental tools continue to improve and quantitative data becomes richer, oscillator theory remains a powerful language for exploring the temporal structure of living systems. The challenge ahead is to refine these models, capturing not only regularity, but also variability, context‐dependence, and failure modes, so they can help us interpret complex biological rhythms across scales, and ultimately inform interventions in health and disease (Box 5).

Box 5 **Glossary of oscillator theory terms**

***Amplitude relaxation rate***: A parameter quantifying how quickly an oscillator returns to its limit cycle after a perturbation. It distinguishes weak from strong oscillators and influences entrainment dynamics, phase shifts, and robustness to noise.

***Arnold onion***: An extension of Arnold tongue diagrams that incorporates additional parameters such as photoperiod or day length. Arnold onions visualize entrainment ranges across seasonal or environmental conditions and help model adaptation in high‐latitude organisms.

***Arnold tongue***: A diagram depicting regions of stable synchronization or entrainment as a function of frequency mismatch and coupling strength. These regions resemble tongues in parameter space and help visualize entrainment ranges and phenomena such as resonance effects or phase sensitivity.

***Bifurcation***: A qualitative shift in system dynamics triggered by gradual changes in parameters. In oscillator models, bifurcations mark transitions between steady states and rhythmic regimes, such as the onset of oscillations via a Hopf bifurcation.

***Birhythmicity***: The coexistence of two stable rhythmic states under identical conditions. Often observed in nonlinear systems, birhythmicity can lead to abrupt transitions and hysteresis and has been modeled in circadian and vocal fold oscillators.

***Chronotherapy***: A therapeutic approach that aligns medical treatment with the body's circadian rhythms to enhance efficacy and reduce side effects. By timing drug administration to coincide with biological windows of vulnerability or resilience, chronotherapy exploits the temporal organization of physiological processes such as metabolism, immune function, and cell division.

***Coupling***: The interaction between oscillators that influences their timing. Coupling can occur via chemical signals, electrical connections, or mechanical forces, and governs synchronization, coherence, and collective dynamics in oscillator networks.

***Deterministic chaos***: A regime in nonlinear dynamical systems where behavior is aperiodic and highly sensitive to initial conditions, despite being governed by deterministic rules. In biological oscillators, chaos can emerge under strong nonlinear coupling and has been observed in vocal systems and hypothesized in circadian networks under extreme conditions.

***Entrainment***: The alignment of an internal oscillator to an external periodic signal (zeitgeber), resulting in a stable phase relationship. Entrainment is a form of unidirectional coupling and is essential for adapting biological rhythms to environmental cycles.

***Harmonic oscillator***: A system exhibiting damped periodic behavior, characterized by a defined period, amplitude, phase, and a damping rate. In biological contexts, such oscillators require external energy input to sustain rhythms and often serve as models for transient or driven oscillations.

***Hopf bifurcation***: A specific type of bifurcation where a stable fixed point loses stability and gives rise to a limit cycle. This transition underlies the emergence of self‐sustained rhythms in biological systems when feedback delays and nonlinearities reach critical thresholds.

***Hysteresis***: A memory effect in dynamical systems where the system's response to a changing parameter depends not only on its current value but also on the direction of change. This means that the transition between states can occur at different parameter values depending on whether the parameter is increasing or decreasing. In biological rhythms, hysteresis can help explain regime shifts (such as transitions between two rhythmic states, or between rhythmic and arrhythmic behavior) in response to environmental cues. A memory effect in dynamical systems where the transition between states depends on the direction of parameter change. In biological rhythms, hysteresis can explain regime shifts in response to environmental cues.

***Limit cycle***: A self‐sustained rhythmic trajectory in a nonlinear dynamical system. Limit cycles persist after transient disturbances and are maintained by internal energy sources and delayed feedback. They act as attractors in phase space and are central to modeling autonomous biological rhythms such as circadian clocks.

***Oscillator networks***: Systems composed of multiple interacting oscillators. As coupling increases, networks exhibit enhanced amplitude, reduced phase dispersion, and can transition from incoherence to complete synchronization. Heterogeneities in periods and amplitudes can produce phase waves and complex spatiotemporal patterns.

***Phase***: The position of an oscillator within its cycle, typically expressed as an angle or fraction of the period (from 0 to 2π, or 0 to 1). Two oscillators may have identical periods but different phases if their cycles are shifted in time. In biological terms, phase describes the timing of a rhythmic event (such as peak gene expression or activity onset) relative to a reference point (e.g., light onset or another oscillator). When no external reference is available, chronobiologists often refer to the peak phase (acrophase) of a self‐sustained oscillator as its phase, though this convention should always be specified in figure legends or text.

## Author contributions

HH and MO conceived the study; HH and MO designed the structure and scope of the review; HH supervised the study; MO, HH and CE researched the literature, wrote the manuscript, and contributed to figure design and revisions. All authors read and approved the final version of the manuscript.
